# Determinants of oral health status: an ecological study in Iran

**DOI:** 10.1186/s12903-023-03557-z

**Published:** 2023-11-22

**Authors:** Bahareh Tahani, Alireza Akbarzadeh Baghban, Ali Kazemian

**Affiliations:** 1 Department of Oral Public Health, Dental Research Center, Dental Research Institute, Dental School, Isfahan University of Medical Sciences, Isfahan, Iran; 2 Proteomics Research Center, Department of Biostatistics, School of Allied Medical Sciences, Shahid Beheshti University of Medical Sciences, Tehran, Iran; 3 Department of Community Oral Health, Faculty of Dentistry, Mashhad University of Medical Sciences, Mashhad, Iran

**Keywords:** Ecological study, Oral health, Social determinants of Health, Epidemiologic factors

## Abstract

**Objectives:**

The aim of this ecological study was to assess the association between behavioral, social position, circumstance factors, and caries experience in 35- to 44-year-old adults in Iran at a provincial level.

**Materials and methods:**

The data from the 2011 Iranian Oral Health Survey were obtained from all 31 provinces across Iran on the population level. Oral health status was measured as the number of decayed, missing (MT), and filled (FT) teeth and the percentage of the population who were edentulous. Data were also gathered from each province on the percentage of smokers (Non-Communicable Diseases Risk Factors Surveillance Provincial Report 2009), per capita consumption of free sugars, concentration of fluoride in the drinking water (National and Sub-national Burden of Disease (NASBOD) Survey), number of dentists per 10,000 people, mean years of schooling of adults, expected years of schooling of children, life expectancy at birth and Gross National Income (Integrated Public Use Microdata Series, Global Data Lab). The data were analyzed using simple and multiple linear regression (α = 0.05).

**Results:**

Mean DMFT was positively associated with the percentage of smokers (B = 0.01 95%CI 0.01–0.14), and negatively with fluoride concentration (B =-2.6 95%CI -4.3- -0.96). The edentulousness percentage was positively associated with smoking (B = 0.2 (with 95%CI: 0.07–0.37) and negatively with mean years of education (B =-1.08 (with 95%CI: -2.04- -0.12). DT was associated with expected years of schooling (B =-0.6 (with 95%CI: -1.07- -0.17), negatively. Mt was negatively associated with life expectancy (B =-0.5 (with 95%CI: -1.1- -0.007), fluoride concentration (B =-3.4 (with 95%CI: -4.5- -1.5) and number of dentists per 10,000 people (B =-0.4 (with 95%CI: -0.8- -0.01). Mean Years of Schooling (B = 0.5 (with 95%CI: 0.2–0.8) and number of dentists per 10,000 people (B =-0.62 (with 95%CI: 0.51 − 0.48) were positively in associated with FT.

**Conclusions:**

The present findings indicate that there were differences in the oral health measures and their social determinants among the provinces of Iran. Regarding the limitations of the study especially the limitation of the number of independent variables, it seems, this discrepancy could be better explained by social variables of the provinces such as income than by environmental factors.

## Introduction

Oral conditions have been considered as a major public health problem that affected approximately 3.5 billion cases worldwide in 2017. According to the Global Burden of Disease estimates, the highest prevalence of dental disease is mainly in low and middle-income countries [1]. The DMFT of 35–44 years old adults in Iran, as a middle-income country in the Eastern Mediterranean region, is about 13.2 ± 0.16 and the prevalence of edentulousness is about 4% [2]. However, obvious disparity in the oral health status of people has been shown among different provinces in Iran. The findings of a recent study among 128,813 adults aged 35 suggested that DMFT was mainly concentrated among the socioeconomically disadvantaged ones [3]. Inequality in the number of nonreplaced extracted teeth also has been reported among adult populations in Iran (greater prevalence among participants who had less than 12 years of schooling and those in the poorest quintile regarding wealth index) [4]. It was claimed that educational background, affects nonmaterial characteristics such as oral health literacy, oral health behaviors (like dietary and tooth brushing habits) and health service utilization frequency and patterns [4, 5].

On the other hand, the role of structural determinants including economic, social and welfare policies in establishing social hierarchies and influencing the socioeconomic status of individuals within societies has been highlighted in the WHO conceptual framework for action on the social determinants of health [6]. It is underlined that structural factors can impact health through intermediate determinants such as housing and working conditions, social capital, psychosocial factors, social support, and access to health care [7]. It has been reported that there are substantial social disparities in the distribution of dental services across the country and generally dentists are more likely to be located in the provinces with better social ranks [8].

Over time, behavioral sciences have expanded our understanding of oral health beyond “disease” to a broader biopsychosocial concept of oral health and to the role of lifestyle or behavioral determinants, known as proximal risk factors [9, 10]. Diet high in free sugars and behaviors such as tobacco smoking, oral hygiene, and use of dental services have been identified as key behaviors that are critical for oral health [11]. Good oral hygiene is key to preventing and maintaining periodontal health while its role in preventing dental caries is less conclusive [12]. Regular dental attendance could be important in identifying oral health problems at early stages, managing them conservatively, and improving the adherence to preventive care [13]. Sugar is increasingly recognized as a global public health issue that is related to both social and commercial determinants of health. Based on the WHO updated guidance on the sugar intake for adults and children, the intake of free sugars throughout the life course is currently high, and it should be reduced to less than 10% of total energy intake. However, WHO suggests a further reduction of the intake of free sugars to below 5% of total energy intake as a conditional recommendation [14]. Smoking affects oral disease locally and systemically through vasoconstriction caused by nicotine leading to the enhancement of subgingival anaerobic bacteria colonization. Recent research also reported that tobacco smoke alter the bacterial surface and promote biofilm formation [15].

Recognition of the social determinants of oral health inequalities has crucial implications for strategy development at the local, regional and national levels [16]. Based on the current evidence, it is emphasized that future action on tackling oral health inequalities requires a reorientation of oral health policy away from a mere focus on changing oral health behaviors to further actions on the common social determinants [17]. However, as oral health surveys do not usually provide information on the role of socioeconomic factors, other study designs such as ecological studies are usually needed as complementary analyses. Therefore, this ecological study aimed to assess the association between different relevant factors and caries experience in 35- to 44-year-old adults in Iran.

## Materials and methods

**Fig. 1:**
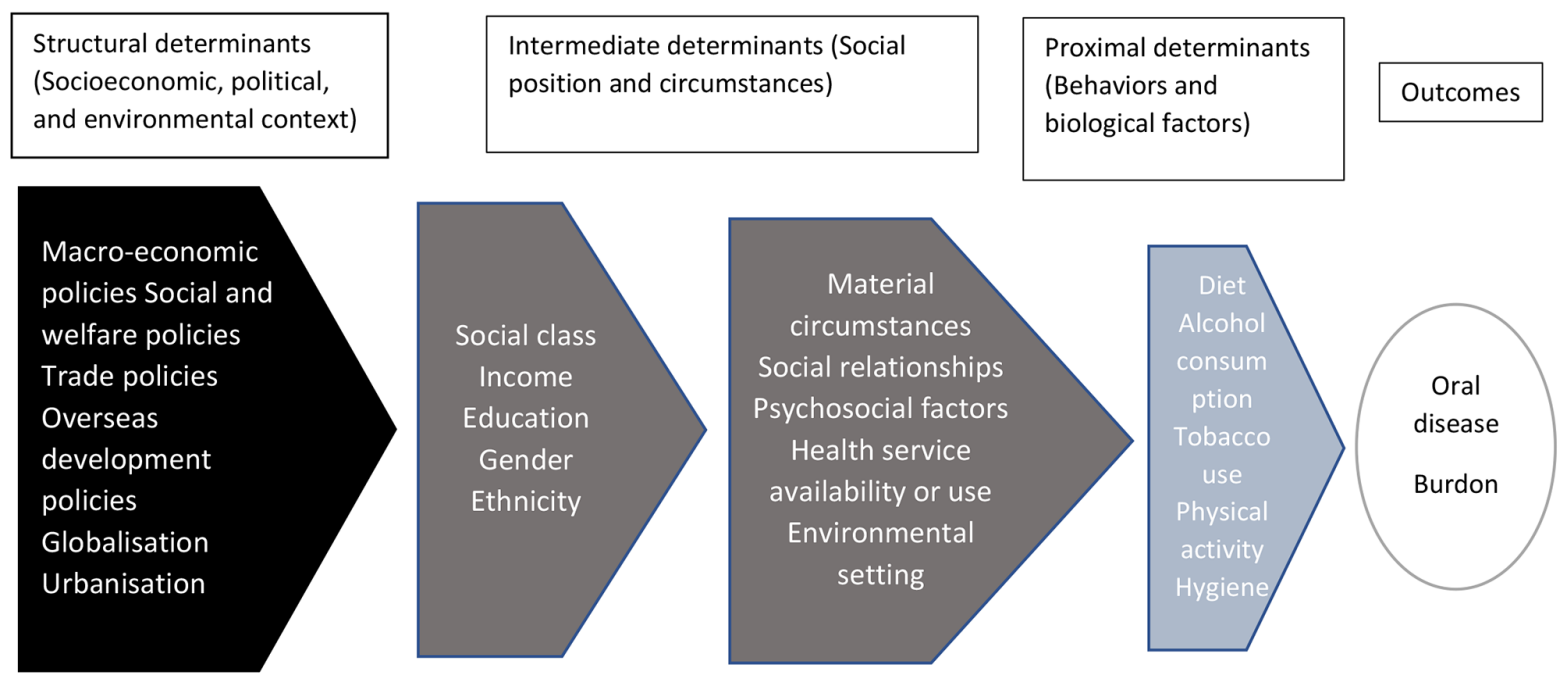
Guiding framework for social and commercial determinants of oral diseases

The framework developed by Peres et al. [18], based on the Watt and Sheiham’s framework of social determinants of oral health [9], was used as the guiding map in order to decide which determining factors be considered in the analysis (Fig. 1). The unit of analysis was the provinces. No sampling method was used because the data of all 31 provinces were retrieved. All of the recruited data were published at the province-level.

### Oral health status

Mean DMFT and percentage of edentulous population were used as outcome variable. National statistics on dental caries experience for 35- to 44- year-old adults were obtained from the latest published National Oral Health Survey conducted during the 2010–2011 period, which was based on the WHO Oral Health Survey basic method [19]. This survey was undertaken by the Oral Health Bureau in the Ministry of Health and Medical Education.

### Behavioral factors

To examine the association of the behavioral determinants with outcome measures, the diet and smoking status were chosen; the provincial level of percentage of smokers among the 35–44 year old population was obtained from the Non-Communicable Diseases Risk Factors Surveillance Provincial report 2009 [20]. The provincial per capita consumption of free sugars was also found in the 2010 provincial report of the free sugar and other foods consumption in Iran [21].

### Environmental factor

The concentration of fluoride in drinking water was considered as one of the (environmental) determinants with the data obtained from the report of the provincial concentration of 15,039 water samples taken from rural and urban water resources in 31 provinces of Iran in 2010, including wells, springs, rivers, water reservoirs, water distribution, and aqueducts—a main source of drinking water in Iran [22].

### Health service availability factor

To estimate the role of health service availability, the provincial-level data on the number of dentists per 10,000 people practicing in public and private sectors in each province based on the 2010 report of Iran Medical Council was used [23].

### Socio-economic factors

To consider the socio-economic status at provincial level, the indices of Subnational Human Development Index (SHDI) [24] were used. SHDI is an average of the subnational values for three main dimensions of education, health and standard of living which are measured through the following indicators:

‘Mean years of schooling of adults aged 25+’ and ‘expected years of schooling of children aged 6’ for measuring the education level; ‘life expectancy at birth’, as the major indicator of health, and ‘Gross National Income per capita’ (PPP, 2011 US$) for measuring the standard of living.

The provincial data set of the year 2011 for Iran was elicited from the website of Global Data Lab (GDL) [25].

#### Statistical analysis

Since both the outcome and the independent variables were measured on a continuous scale, data was analyzed using simple and multiple linear regression to estimate the association of independent (explanatory) factors with the DMFT index and its components (DT, MT and FT), as well as the edentulousness percentage, using SPSS (IBM SPSS Statistics 22). The level of significance was considered as 0.05.

## Results

The proposed data set was obtained for all 31 provinces in Iran, except sugar consumption, which was available only for 20 provinces at the time of the study.

Table 1 presents descriptive statistics for the outcome and independent variables used in this study. Regarding the dental caries, the means of DT, FT and MT were 4.4 ± 1.2 (1.8–6.9), 2.1 ± 1.1 (0.3–5.1) and 7.3 ± 1.8 (2.8–10.8), respectively. In other words, MT defined about 52.3% of the total DMFT. The proportions of the FT and DT components in defining the total DMFT were 15.5% and 32.2%, respectively.

**Table 1:** descriptive indices of the outcome and independent variables at the province level

Variables	Number of Provinces	Mean ± Std	Minimum-Maximmum among provinces
**Outcome variables**			
DMFT	31	13.8 ± 1.8	8.6–18.5
Edentulousness ^*^	31	4.4 ± 3.6	0.1–13.4
**independent variables**			
life expectancy	31	74.3 ± 0.9	71.2–75.6
Gross National Income per capita	31	18.1 ± 2.6	10.9–24
Expected years of schooling	31	13.5 ± 0.8	10.5–14.7
Mean years of schooling of adults	31	8.6 ± 1.1	5.9–11.2
Smoker percentage	31	30.6 ± 8.2	15.9–46.6
Number of working dentists per 10,000 people	31	2.1 ± 1.3	0.8–7.9
Concentration of Fluoride(mg/l)	31	0.5 ± 0.3	0.25–1.86
Sugar consumption per capita in Kg	20	15.1 ± 4.5	8.9–25.3

Note-*, Edentulousness has been reported as mean of prevalence in each province

The distribution of DMFT and edentulousness were normal (testing by Shapiro-Wilk normality test) therefore we used linear regression. The Shapiro-Wilk statistics for DMFT and mean percentage of edentulousness were 0.94 (p = 0.09) and 0.93 (p = 0.06), respectively. The results of the simple linear regression for DMFT and edentulousness are shown in Table 2. There was a positive correlation between the mean DMFT and the percentage of smokers (p = 0.003, r = 0.5, *B* = 0.11 with 95%CI: 0.04–0.18), while a negative correlation was found between DMFT and the fluoride concentration of the provinces (p < 0.001, r=-0. 6, *B*=-3.03 with 95%CI: -5.1- -1.61).

**Table 2:** Simple regression analysis of predictor factors on DMFT mean and edentulousness percentage in all 31 provinces

Outcome measure	Independent Variables	*B(SE)* (Unstandardized coefficient and standard error)	r	95% CI for *B*	R square in percentage
DMFT	Life expectancy	-0.43(0.33)	-0.24	-1.1- 0.23	5
	Mean Years of Schooling	-0.31(0.32)	-0.16	-0.96- 0.36	2
	Expected years of schooling	-0.00(0.39)	-0.00	-0.8- 0.79	0.1
	GNI	-0.01(0.13)	-0.02	-0.27 -0.24	0.1
	Sugar Consumption	0.06(0.10)	0.14	-0.15-0.28	2
	Percentage of Smokers*	0.11(0.03)	0.51	0.04–0.18	26
	Fluoride concentration **	-3.03(0.83)	-0.64	-5.1- -1.61	36
	Dentists per 10,000 people	-0.04(0.26)	-0.02	− 0.57- 0.49	0.1
Edentulousness	Life expectancy	-1.01(0.65)	-0.27	-2.43-0.33	7
	Mean Years of Schooling	-1.01(0.63)	-0.28	-2.31-0.28	8
	Expected years of schooling	-0.18(0.78)	-0.04	-1.7-1.4	0.2
	GNI	0.12(0.25)	0.09	-0.39-0.64	0.8
	Sugar Consumption	0.12(0.13)	0.19	-0.16-0.39	3
	Percentage of Smokers*	0.24(0.07)	0.55	0.1–0.38	30
	Fluoride concentration *	-4.17(1.95)	-0.37	-8.1- -0.17	14
	Dentists per 10,000 people	-0.43(0.51)	-0.10	-1.4-0.64	2

Note- *= P-value < 0.05, **= P-value < 0.001

Similarly, regarding edentulousness, the only factor with a positive association was smoker percentages (p < 0.001, r = 0.5, *B* = 0.24 with 95%CI: 0.1–0.38). A negative correlation was reported also with fluoride concentration (p = 0.04, r=-0.3, *B*= -4.17 with 95%CI: -8.1- -0.17).

The results of the simple regression for DT, MT and FT are shown in Table 3. Regarding DT, the factors with a negative correlation were GNI (p = 0.03, r=-0.39, *B*=-0.17 with 95%CI: -0.32- -0.02), mean years of schooling (p = 0.04, r= -0.37, *B* = − 0.42 with 95%CI: -0.82- -0.02), and expected years of schooling (p = 0.003, r=-0.51, *B* = − 0.69 with 95%CI: -1.13- -0.25).

**Table 3:** Simple regression of associations between intermediate and proximal factors with components of DMFT

Outcome measure	Independent Variables	*B* (SE) (unstandardized coefficient and standard error)	r	95% CI for *B*	R square in percentage
DT	Life expectancy	-0.15(0.21)	-0.12	-0.59-0.29	2
	Mean Years of Schooling*	-0.42(0.19)	-0.37	-0.82-0.02	13
	Expected years of schooling **	-0.69(0.21)	-0.51	-1.13- -0.25	26
	GNI*	-0.17(0.07)	-0.39	-032- -0.02	15
	Sugar Consumption	0.07(0.05)	0.35	-0.04-0.18	9
	Percentage of Smokers	-0.04(0.02)	-0.31	-0.09-0.01	8
	Fluoride concentration	-0.01(0.67)	-0.01	-1.41-1.32	0.1
	Dentists per 10,000 people	-0.02(0.16)	-0.31	-0.56-0.09	6
MT	Life expectancy*	-0.77(0.32)	-0.41	-1.41- -0.12	17
	Mean Years of Schooling*	-0.85(0.29)	-0.47	-1.49- -0.24	22
	Expected years of schooling	-0.42(0.39)	-0.19	-1.20-0.38	3
	GNI	-0.14(0.13)	-0.21	-0.41-0.11	4
	Sugar Consumption	0.08(0.09)	0.20	-0.12-0.28	4
	Percentage of Smokers*	0.10(0.04)	0.45	0.02–0.17	21
	Fluoride concentration *	-3.08(0.88)	-0.55	-4.92- -1.2	30
	Dentists per 10,000 people	-0.41(0.25)	-0.31	-0.95-0.07	9
FT	Life expectancy*	0.41(0.18)	0.38	0.03–0.79	14
	Mean Years of Schooling**	0.75(0.14)	0.71	0.47–1.02	50
	Expected years of schooling **	0.78(0.18)	0.63	0.42–1.11	39
	GNI*	0.21(0.06)	0.53	0.08–0.34	28
	Sugar Consumption	-0.06(0.05)	-0.25	-0.17-0.05	6
	Percentage of Smokers	0.04(0.02)	0.29	-0.01-0.08	8
	Fluoride concentration	-0.14(0.60)	-0.04	-1.37-1.09	0.2
	Dentists per 10,000 people**	0.52(0.11)	0.59	0.35–0.81	48

Note- *= P-value < 0.05, **= P-value < 0.001

Considering FT, factors including life expectancy (p = 0.03, r = 0.38, *B* = 0.41 with 95%CI: 0.03–0.79), mean years of schooling (p < 0.001, r = 0.71, *B* = 0.75 with 95%CI: 0.47–1.02), expected years of schooling (p < 0.001, r = 0.4, *B* = 0.75 with 95%CI: 0.42–1.1), number of dentists per 10,000 people (p < 0.001, r = 0.6, *B* = 0.52 with 95%CI: 0.35–0.8), and GNI (p < 0.001, r = 0.53, *B* = 0.21 with 95%CI: 0.1–0.38) were in association with the mean number of the filled teeth positively.

The factors with a negative correlation with MT included life expectancy (p = 0.021, r=-0.41, *B*= -0.77 with 95%CI: -1.41- -0.12), mean years of schooling (p = 0.007, r=-0.47, *B*= -0.85 with 95%CI: -1.49- -0.24), and fluoride concentration (p = 0.002, r=-0.55, *B*= -3.08 with 95%CI: -4.9- -1.2). Percentage of smokers (p = 0.01, r=-0.45, *B* = 0.1 with 95%CI: 0.02–0.17) was positively associated with MT.

Multiple linear regression analysis was used to identify the best indicators of mean DMFT and edentulousness using a stepwise technique (Table 4). The fluoride concentration (*B*= -2.6 with 95%CI: -4.3 - -0.94) and the percentage of smokers (*B* = 0.08 with 95%CI: 0.01–0.14) were the strongest indicators for DMFT and remained in the model. This model explained 47% of the variation (R^2^) in DMFT mean across provinces. The collinearity of indicators was checked by tolerance and Variance Inflation Factor (VIF) that were 0.87 and 1.1, respectively, for both variables indicating low level of collinearity.

**Table 4:** Stepwise multiple linear regression analysis of predictor factors on DMFT and its components mean and edentulousness percentage in all 31 provinces

Dependent and independent variables	Unstandardized Coefficients	Sig.	95.0% Confidence Interval for *B*	
	*B(*SE*)*		Lower Bound	Upper Bound
**Edentulousness%**				
Smoking population%	0.22	0.007	0.07	0.37
Mean years of schooling	-1.08	0.029	-2.04	− .12
**DMFT**				
Smoking population%	0.01	0.027	0.01	0.14
Fluoride concentration	-2.61	0.004	-4.35	-0.96
**DT**				
Expected years of schooling	-0.62	0.009	-1.07	-0.17
**MT**				
Fluoride concentration	-3.04	0.001	-4.57	-1.51
Life expectancy	-0.59	0.031	-1.12	-0.07
Dentists per 10000 people	-0.42	0.041	-0.89	-0.01
**FT**				
Mean years of schooling	0.56	0.002	0.24	0.89
Dentists per 10000 people	0.24	0.042	0.01	0.48

Note-The R squares for percentage of edentulousness, DMFT, DT, MT and FT were 44%, 47%, 34%, 67%, 78%, respectively

Regarding the edentulousness percentage as outcome measure, percentage of smokers and mean years of schooling were remained as the main indicators. The tolerance and VIF scores were 0.98 and 1.01, respectively. These two indicators explained 44% of the variation in percentage of the edentulousness. The indicator factor for DT was expected years of schooling, which explained 34% of the variation of DT. The factors with strongest association with MT were fluoride concentration, life expectancy and dentist per 10,000 people that had the tolerance of 0.95 to 0.89 and VIF of 1.04 to 1.1 and explained 67% of the MT variation. Regarding FT, mean years of schooling and dentist per 10,000 people remained in the model that explained 78% of the variation of the FT. The tolerance and the VIF scores were 0.52 and 1.9, respectively.

## Discussion

In our ecological study, the association of the determinants of oral health at provincial level was assessed. Ecologic studies are valuable study designs that may be promising in casting light on etiologic relationships. However, they could not demonstrate the existence of true associations conclusively [26]. The studies on aggregated data, as our ecological study, could benefit from some critical advantages over the individual-based studies [27], such as explaining the contextual effects on the prevalence of disease, lower cost, analytical simplicity and ethical appropriateness, plus the possibility of measuring the population-level exposures, e.g., the Human Development Index of a region. Our study also benefited from the availability of the national surveys data with the similar measurement scales of all provinces of Iran. The data made it possible to compare variables vigorously across the provinces.

However, it is critical to avoid the ecological fallacy as the main limitation of inference from the ecological studies. It refers to the impossibility of inferring the results obtained at the population level to the individual level [26]. Furthermore, in the current ecological study, the number of independent variables included in the analysis was limited and we were not able to consider all the proposed determinants based on the guiding frameworks. Specially, variables such as gender, urban/rural distribution, age groups and cumulative life-time effects of exposure to risks, ethnic variation within the provincial data, alcohol consumption, and oral health behavioral factors were not available. Besides, data for sugar consumption was only available from 20 provinces. Furthermore, the data might be old. However, as the last national survey among adults was held in 2011, we had to collect the other data for the same time period. Another limitation was that periodontal diseases were not assessed in the national survey and accordingly was not analyzed in our study. Availability of such data, might be precious for evaluating the proposed paths of determinants such as smoking and outcomes such as edentulousness or MT.

Based on the results of simple regression analysis, the main two outcomes, i.e. DMFT and edentulousness, were associated with the percentage of smokers (positively) and the fluoride concentration (negatively). This finding was reconfirmed in the multiple regression model. Factors such as GNI, years of schooling and expected years of schooling significantly explained the variation of DT and FT components across provinces. In particular, based on the results of multiple regression models, the FT mean was highly and positively associated with the education level and the proportion of dentists to population.

It was notable that while the lower mean years of schooling was not associated with the higher aggregate disease experience (i.e., DMFT), it was associated with the type of the received dental treatment, either filling or extraction. The same results were also reported in the study conducted by Mejia et al. on Australian adults [28], where social gradients in caries were evident but particularly notable in Missing and untreated Decay. Their findings, thus, indicated that social gradients for dental caries could have a greater effect on how the disease was treated, as compared to lifetime disease experience. The number of dentists per 10,000 people was another factor with a strong and direct impact just on FT. Since the per capita number of dentists was higher in the more affluent provinces with higher GNI, this finding indicated that dentists tended to be located in more advantaged parts of the country, while residents of disadvantaged provinces who faced more difficulty in meeting their basic dental needs had less access to dental services. It could serve as a good example of the inverse care law and might enforce inequities in the prevalence of untreated dental caries [29].

According to the results of the national survey, about 5% of the population in Iran could be considered edentulous and about 52.3% of the total DMFT was defined by the missing teeth (MT). Tooth loss is a complex outcome considered as an effective marker of a population’s oral health, reflecting both the accumulated individuals’ history of dental disease and the characteristics of the oral health care system [30]. In a systematic review conducted to assess the main reasons for extractions of permanent teeth in adults, it was suggested that dental caries and periodontitis were the main indications for dentists to perform dental extractions [31].

The association of tooth loss with lower income and schooling, as reported in different other studies [32], could be explained, at least partly, by the fact that poorer and less educated individuals have less access to dental services and oral hygiene products [33]. Also, they usually consume more sugar [34] and brush their teeth less frequently. On the other hand, people with higher schooling have more dental appointments and higher self-perception about the state of their oral health condition and the need for dental treatment [35]. The reasons for more extractions among people with lower schooling, therefore, could be the result of extensive untreated disease or the tendency to choose the lowest cost services [36].

Although the prevalence of complete tooth loss in our study population was not considerable (about 5%), the impact of tobacco consumption and low exposure to systemic fluoride on edentulousness was visible. The association of tobacco consumption with MT and the eventual complete tooth loss might be mediated by the correlation of tobacco and periodontal disease as the main risk factor for tooth loss in adults [37]. However, since the focus of our study was mainly on dental caries, a deeper analysis of the mediation by periodontal conditions would be helpful in the future studies.

Low exposure to systemic fluoride has been recognized as one of the factors contributing to caries progression. In a study undertaken on Australian younger adults, the lifetime fluoridation exposure led to the significantly and substantially lower DMFT and number of filled teeth [38]. Griffin et al., in their systematic review, also concluded that water fluoridation reduced caries by 27% in adults [39]. Another study compared the state of teeth in young adults who had consumed fluoridated water from birth to 5–8 year of age with the subjects who had non-fluoridated water, revealing that systemic fluoride could lead to less missing teeth and lower progressed dental caries [40]. In another ecological study conducted by Ekstrand et al., fluoride level of the drinking water was a significant variable explaining the variations in mean DMFS among municipalities in Denmark [41]. The strong association of lower missing teeth and DMFT with a higher concentration of drinking water fluoride in our study might be explained by the same effect as the progressed dental caries could usually result in extraction.

Findings of this study, in line with those of the ecological study of Antunes et al. [42], which was carried out at district level, showed that the more distant and impoverished areas had a higher level of dental caries prevalence. In Addition, the ecological study by Pattussi et al. [43] concluded that the social inequalities were associated in a significant way with the inequalities found in the distribution of cavities.

To conclude, there was an apparent discrepancy among the provinces of Iran in the oral health measures and their social determinants. The social gradient was evident, particularly in the distribution of “decayed” and “filling” teeth. In light of the study’s limitations, particularly the constraint on the number of independent variables, it appears that the observed disparity can be more effectively elucidated by social determinants such as the Human Development Index and provincial income levels rather than environmental factors. Nevertheless, in order to arrive at a more definitive conclusion and ascertain the precise extent to which each factor contributes to the explained variance, it is imperative to conduct more comprehensive investigations encompassing a broader range of variables. Average years of schooling could explain mean number of decayed, missing, and filling teeth, separately, but not the aggregate mean DMFT of the population- indicating the limitation of DMFT as the most commonly used dental indicators.

## Data Availability

The data that support the findings of this study are available on request from the corresponding author.
